# Frequent expression of new cancer/testis gene D40/AF15q14 in lung cancers of smokers

**DOI:** 10.1038/sj.bjc.6600328

**Published:** 2002-06-05

**Authors:** M Takimoto, G Wei, H Dosaka-Akita, P Mao, S Kondo, N Sakuragi, I Chiba, T Miura, N Itoh, T Sasao, R C Koya, T Tsukamoto, S Fujimoto, H Katoh, N Kuzumaki

**Affiliations:** Division of Cancer Gene Regulation, Research Section of Disease Control, Institute for Genetic Medicine, Hokkaido University, Sapporo, Hokkaido, Japan; Department of Medical Oncology, Graduate School of Dental Medicine, Hokkaido University, Hokkaido, Japan; Department of Surgical Oncology, Graduate School of Dental Medicine, Hokkaido University, Hokkaido, Japan; Department of Obstetrics and Gynecology, Graduate School of Medicine, Graduate School of Dental Medicine, Hokkaido University, Hokkaido, Japan; Oral Diagnosis and Oral Medicine, Department of Oral Patho-Biological Science, Graduate School of Dental Medicine, Hokkaido University, Hokkaido, Japan; Department of Urology, Sapporo Medical University, Sapporo, Hokkaido, Japan

**Keywords:** D40, testis, cancer/testis (CT), AF15q14, lung cancer, smoking

## Abstract

We found a significant correlation between lung cancer in smokers and the expression of a human gene, D40, predominantly expressed in testis and cancers. In an attempt to clone a novel human gene, we screened a cDNA library derived from a human B cell line and obtained a cDNA clone that we refer to as D40. A search for public databases for sequence homologies showed that the D40 gene is identical to AF15q14. D40 mRNA is predominantly expressed in normal testis tissue. However, this gene is also expressed in various human tumour cell lines and primary tumours derived from various organs and tissues, such as lung cancer. We examined the relationship between D40 expression and clinico-pathological characteristics of tumours in primary lung cancer. D40 expression did not significantly correlate with either histological type or pathological tumour stage. However, D40 expression was observed more frequently in poorly differentiated tumours than in well or moderately differentiated ones. Furthermore, the incidence of D40 expression was significantly higher in tumours from patients who smoke than in those from non-smokers. D40/AF15q14 is the first gene in the cancer/testis family for which expression is related to the smoking habits of cancer patients.

*British Journal of Cancer* (2002) **86**, 1757–1762. doi:10.1038/sj.bjc.6600328 www.bjcancer.com

© 2002 Cancer Research UK

## 

Tumour cells often express genes that are otherwise expressed in normal cells at very low levels if at all (Bishop, 1987). Such aberrantly expressed genes include proto-oncogenes and those involved in tissue-specific differentiation. The expression of these genes in tumour cells probably contributes to the malignant phenotypes and the recognition of tumour cells by the host immune system (Boon *et al*, 1997).

The testis, an essential organ of the male reproductive system, produces spermatozoa in which genetic information is stored within the haploid genome (Bloom and Fawcett, 1994). Of the dozens of genes that are expressed in the testis, some are expressed in a testis-specific manner related to spermatogenesis. Whereas most testis-specific genes are either never or very rarely activated in tumours (De Smet *et al*, 1997), recent studies have identified a class of genes that is expressed both in the normal testis and in cancerous tissue. Some of them elicit an immune response in hosts and are called cancer/testis (CT) antigen (Boon *et al*, 1997; Chen and Old, 1999). Others, for which the antigenicity remains unknown, are expressed in similar manner to CT. Some of these display sequences homology to CT and are expressed not only in testes but also in other male and female reproductive organs (Brinkmann *et al*, 1998). Overall, these may all be referred to CT family genes or CT genes. Their potential application in the diagnosis and immunogene therapy of cancer has been extensively studied (Boon *et al*, 1997; Chen and Old, 1999). However, little is known about either clinico-pathological features of their expression in tumours or their physiological functions. This information is very important in characterisation of CT genes and when considering immunotherapy with these genes. Understanding the clinico-pathological characteristics of their expression would facilitate determination of which CT gene product should be selected as tumour targets for therapy.

In this study, we report on a human gene, D40, that is primarily expressed in normal testis tissues. Initially, we searched for proteins that interact with the transcription factor GCF using the yeast two-hybrid system (Kageyama and Pastan, 1989; Chien *et al*, 1991). After we obtained a cDNA clone, D40, that binds to GCF, we later discovered that the reported GCF cDNA clone is chimeric, consisting of two different cDNA fragments. One was derived from the GCF2 transcription factor and the other was from unrelated gene product with unknown function (Reed *et al*, 1998; Takimoto *et al*, 1999). D40 protein binds to the latter. A sequence homology search revealed that D40 is the same gene as AF15q14 which was recently identified as one of the genes that fuses with an oncogenic gene MLL (mixed lineage leukaemia) in acute leukaemia (Hayette *et al*, 2000). While the D40 gene is barely expressed in normal tissues except for the testis, it is expressed frequently in various human cancer cell lines and primary tumours derived from different tissues and organs, suggesting that D40 is a novel member of the CT family. Furthermore, the incidence of D40 expression is significantly higher in poorly differentiated primary lung cancer and in cancers from patients with a smoking habit. To our knowledge, D40/AF15q14 is the first gene in the CT family for which expression is significantly related to smoking habits of the patients with cancer.

## MATERIALS AND METHODS

### Cultured cell lines, tumour samples, and patients

Cell lines used in this study are described in Table 1

**Table 1:**
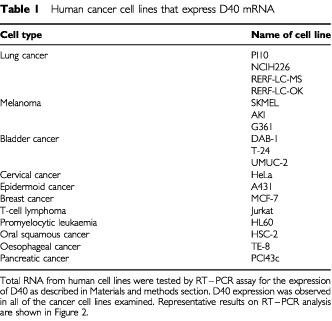
Human cancer cell lines that express D40 mRNA

. The cultured cells were maintained in RPMI 1640 or DMEM with 10% foetal calf serum and 0.3 mg ml^−1^ L-glutamine. Cells were cultured at 37°C in a 5% CO_2_ atmosphere. Primary tumour samples obtained from surgical operations were quickly frozen in liquid nitrogen and stored at −80°C until RNA isolation. Experiments on surgical samples were performed after obtaining written informed consent from the patients. Sections of tumour specimens were reviewed by pathologists. Lung tumour specimens were histopathologically diagnosed according to the 1981 World Health Organization classification (WHO, 1982). The postsurgical pTMN stage was determined according to the guidelines of the American Joint Committee on Cancer (Bearhs *et al*, 1992). Smokers analysed in this study were current smokers, including individuals who stopped smoking 3 months before surgery. Non-smokers were those who had never smoked.

### cDNA cloning and DNA sequencing of D40

The carboxy terminal two-thirds of the coding region of the previously described transcription factor GCF (Kageyama and Pastan, 1989) was subcloned into pGBT-9 (Clontech) and the resultant plasmid was used in yeast two-hybrid screening with the yeast strain Y153 and a cDNA library of a human B cell line (Fields and Song, 1989; Chien *et al*, 1991; Durfee *et al*, 1993). Out of 1.2 million yeast clones screened, nine were positive for both histidine and LacZ phenotypes. After confirming that one of the clones possessed binding specificity, we determined the nucleotide sequences. We screened a cDNA library from a human promyelocytic leukaemia HL60 cell line for longer clones using this clone. Rapid amplification of cDNA ends (RACE) (Frohman *et al*, 1988) was performed with the D40-specific primers and the protocol provided with a commercial 5′/3′ RACE kit (Boehringer/Roche). For DNA sequencing, plasmid DNAs prepared with a commercial column (Qiagen) served as templates in cycle sequencing reactions performed using dideoxy chain termination (Sanger *et al*, 1977) Sequence were analysed using fluorescence DNA sequencers. Public databases on the Internet were searched for sequence homology.

### RNA isolation

Total RNA from cell lines and tumour samples was isolated and purified by the acid-guanidinium-phenol chloroform method (Chomczynski and Sacchi, 1987). Approximately 1×10^7^ cells or 80 mg of tumour tissues were homogenised in 2 ml of Trizol reagent (Life Technology) to purify the RNA.

### Northern blot analyses

Northern blot analyses were performed as described (Sambrook *et al*, 1989). Probe DNAs were radiolabelled with α-^32^P-dCTP using a commercial random priming kit (Takara, Kyoto, Japan). Hybridisation proceeded in 50% formamide, 5×SSC, 5×Denhardt's solution and 0.5% SDS at 37°C overnight. Blotted membranes were then washed at progressively higher stringency. The membranes were finally washed in 0.2×SSC containing 0.1% SDS at 48°C. Signals were detected using a BAS2000 (Fuji Film, Tokyo, Japan).

### Reverse transcription polymerase chain reaction (RT–PCR)

Total RNA (1 μg) was reverse-transcribed to cDNA with a reverse primer, MT152 (5′- TCCCATCTTCTGATGTG-3′), and Superscript II RNase H^−^ reverse transcriptase (Life Technology). Amplification was performed with 0.1 μg cDNA, 10 pmol of D40-specific oligonucleotides (forward: MT149; 5′-CACATCCAGTGAGACCA-3′, reverse: MT152), and 0.5 units of Taq polymerase in a total reaction volume of 20 μl with a buffer containing 10 mM Tris-HCl, 1.5 mM MgCl_2_, and 50 mM KCl, pH 8.3. Primers for β-actin were used in the amplification to check the RNA integrity.

### Statistical analysis

Associations between D40 expression and categorical variables were analysed by the χ^2^ test or Fisher's extract test as appropriate (Mehta and Patel, 1983). Associations between D40 expression and age were analysed by the Student's *t*-test. The significance level was *P*<0.05 and all tests were two-sided.

## RESULTS

### Identification and cloning of the human gene D40

In an attempt to clone a novel human gene, we performed two-hybrid screening of the cDNA library derived from a human B cell line using the transcription factor GCF (Kageyama and Pastan, 1989) as bait and were able to obtain a cDNA clone that we called D40. DNA sequencing analyses revealed that the clone had an open reading frame. Using a part of the clone as a probe, a cDNA library from a human leukaemia cell line was screened to obtain longer clones. During the course of cloning, we noticed that the initial D40 clone had a deletion of a nucleotide in the middle of the coding region that leads to an occurrence of a stop codon several bases downstream of the deletion (Takimoto, 1999). As none of the other clones had in-frame stop codons at both ends, we performed rapid amplification of cDNA ends (RACE). We were able to find the clones that had an in-frame stop codon in the 5′ end and those in the 3′ end with 5′ and 3′ RACE analyses, respectively; we called this gene D40. In the later stage of this study, however, it turned out that the sequence of D40 is identical with that of the gene AF15q14 (Hayette *et al*, 2000), except for the 3′ end and several nucleotides. The nucleotide sequence in D40 cDNA revealed in this study was deposited in GenBank/EMBL/DDBJ (accession no: AB022190).

### Expression of D40 mRNA is abundant in testis tissue but not in other normal human tissues

**Figure 1:**
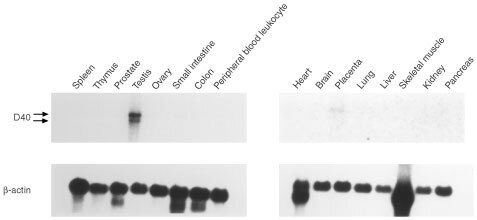
Northern blot analyses of D40 expression in normal human tissues. Northern hybridisations were performed on membranes on which multiple human tissue mRNA samples were blotted as described in the Materials and methods section. Expression of D40 mRNA was observed in the testis tissue sample but not in other normal tissues, except for the placenta sample, which showed trace expression. Arrows indicate the transcripts, 8.5 and 7 kb in length (upper). The membranes were stripped of D40 probe, then rehybridised with β-actin probe to confirm RNA integrity on the membrane (lower).

The expression of the D40 gene in various normal human tissues was examined. Northern hybridisations were performed on membranes on which multiple human tissue mRNA samples were blotted as described in the Materials and methods section. The results revealed that a high level of D40 mRNA expression was observed in testis tissue and a lower level of expression was detected in placenta tissue. A major transcript of about 8.5 kilo base (kb) and a minor one of about 7 kb in size were detected in the testis tissue. However, D40 expression was not significant in any other normal tissues (Figure 1

). With longer exposure of the Northern blot, slight D40 expression was observed in a few tissues, far below that in testis tissue (data not shown). These results indicate that the D40 mRNA is dominantly expressed in testis tissue.

### The D40 gene is expressed in various human cancer cell lines and primary tumours

**Figure 2:**
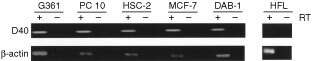
Expression of the D40 gene in human cancer cell lines analysed by the RT–PCR assay. To isolate and purify total RNA, about 1×10^7^ cells of each human cancer cell line were homogenised in 2 ml of Trizol reagent. The RT–PCR assay was performed on the purified RNA as described in the Materials and methods section. Typical results of RT–PCR analyses on D40 expressions in cancer cell lines are shown: G361 (melanoma), PC10 (lung cancer), HSC-2 (oral cancer), MCF-7 (breast cancer), DAB-1 (bladder cancer), HFL (normal fibroblast of lung). RT + or − indicates with or without reverse transcription. The results of β-actin RT–PCR analyses indicate that the RNA integrity was maintained in all samples.

We also observed D40 expression in several tumour cell lines as well as in normal testis tissue. We next examined the mRNA expression of D40 in various human cancers. When we examined D40 expression in different human cancer cell lines, we found that D40 was expressed in the cell lines of four lung cancers, three melanomas, three bladder cancers, one cervical cancer, one epidermoid cancer, one breast cancer, one lymphoma, one leukaemia and tumours derived from the alimentary tract (Table 1). Typical experimental results are shown in Figure 2

.

**Figure 3:**
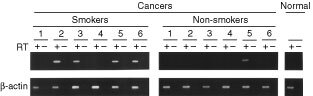
Expression of D40 mRNA in primary human cancer assayed by RT–PCR. Typical experimental results of RT–PCR analyses of D40 expression in primary cancer. D40 mRNA expression was analysed in total RNA from primary lung tumours and from normal lung as described in the Materials and methods. Numbers are individual primary lung tumours from smokers and non-smokers. RT + or − indicates with or without reverse transcription. Levels of β-actin in same RNAs were analysed by RT–PCR to confirm sample integrity.

We then examined D40 expression in human primary tumours. Total RNAs were prepared from frozen samples of human primary tumours obtained from surgical operations. Analyses by the RT–PCR assay on different primary tumours had detected D40 transcripts in significant fractions of primary tumour samples, such as lung cancer, ovary cancer and pancreatic cancer. Typical results of RT–PCR analyses on the primary tumours are shown in Figure 3

. Out of 46 cases of primary lung cancer examined, D40 expression was observed in 19 cases (more than 40%). Several other primary cancers also showed D40 expression with comparable or lower frequencies depending on the types of tumours, such as ovary cancer, 36%; pancreatic cancer, 27%; and cervical cancer, 38% (Table 2

**Table 2:**
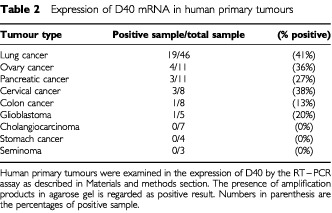
Expression of D40 mRNA in human primary tumours

). These results indicate that the D40 gene is expressed in different human cancers.

### Frequent expression of the D40 gene in poorly differentiated lung tumours and in tumours of smokers

To characterise D40 expression in human primary tumours, lung cancer was chosen in which to examine the potential correlation between D40 expression and clinico-pathological characteristics. The expression of D40 in 46 cases of primary lung cancer was examined in comparison with several characteristic items and the examinations were processed with statistical methods. The results showed that D40 expression has no relation to a patient's age or sex, or to a tumour's histology and pathological stages, such as tumour size and lymph node metastasis. However, the incidence of D40 expression was significantly higher in poorly differentiated tumours than well or moderately differentiated ones (*P*<0.04). Further, significantly higher incidence of D40 expression was observed in primary lung tumours from patients with smoking habits compared with tumours from non-smokers (*P*<0.02) (Table 3

**Table 3:**
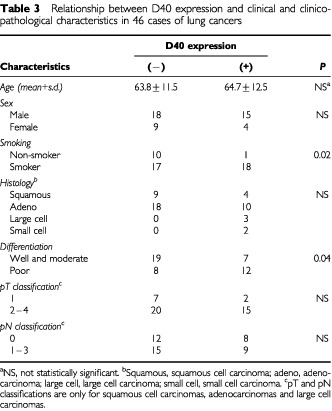
Relationship between D40 expression and clinical and clinicopathological characteristics in 46 cases of lung cancers

).

## DISCUSSION

In this study, we identified human gene D40 that is predominantly expressed in the normal testis tissue, but not in other normal tissues. A sequence homology search revealed that the D40 sequence is the same as that of the AF15q14 gene (Hayette *et al*, 2000). This gene is a partner that fuses with MLL, an oncogenic gene involved in the development of acute leukaemia (Hunger *et al*, 1993; Thirman *et al*, 1994; Hernandez *et al*, 1995; Dimartino and Cleary, 1999). In contrast to normal tissues, D40 was expressed in all human cancer cell lines examined and in several primary human tumours independently of the types and source of the tumours. The incidence of D40 expression was significantly higher in tumours derived from smokers as well as in poorly differentiated tumours.

Most testis-specific genes are either never or very rarely activated in tumours (De Smet *et al*, 1997). However, recent studies, have disclosed a class of genes that is expressed both in the normal testis tissue and in cancer. They are referred as cancer/testis (CT) genes or CT family genes. The expression profile of the D40 gene resembles that of CT genes, suggesting that D40 is also a member of this family. Some of the CT genes elicit immune response in patients with malignancies and these are called CT antigens, constituting several subfamilies of genes, such as MAGE (melanoma antigen) (Van der Bruggen *et al*, 1991; Boon *et al*, 1997; Chen and Old, 1999). They were originally identified as tumour antigens recognised by cytotoxic T lymphocytes or by antibodies in the sera of patients with cancer (Sahin *et al*, 1995; Boon *et al*, 1997; Chen and Old, 1999). Further study is needed to determine whether D40 protein elicits immune responses in patients with cancer.

Very few reports have described the relationship between the CT gene and clinico-pathological characteristic, The size of a primary hepatocellular carcinoma is reportedly larger and the serum α-fetoprotein level is reportedly lower in MAGE-positive than in MAGE-negative patients (Suzuki *et al*, 1999). To our knowledge, no one has reported that expression of a CT gene is significantly correlated to the smoking habits of patients, as this study revealed.

We questioned why a gene expressed in the testis in a restricted manner would be expressed frequently in different human tumours. A clue might be found in the experimental data from primary lung cancer indicating that poorly differentiated tumours and tumours from smokers express D40 at high frequency. Tobacco smoke contains a mixture of highly mutagenic chemicals, such as benzo(a)pyrene and 4-methylnitrosoamine-1-(3-pyridyl)-1-butanone. Benzo(a)pyrene induces point mutations such as G to T transversion (Ruggeri *et al*, 1993) and forms DNA adducts at the major mutational hot spots of the p53 gene in lung cancer (Denissenko *et al*, 1996). The mutagen 4-methylnitrosoamine-1-(3-pyridyl)-1-butanone causes epigenetic alterations of the genome through the modification of DNA methyltransferase activity (Belinsky *et al*, 1996). Cellular genes, including D40, which are barely expressed in normal cells, may be dysregulated so that they are expressed abnormally when exposed to these chemicals in tobacco smoke. Compared with well-differentiated lung tumours, poorly differentiated tumours may be more deviated from the normal state in gene regulation. The poorly differentiated tumour cells may arise through the accumulation of dysregulated gene expressions affected by mutagenic chemicals in tobacco smoke.

In contrast to D40, expression of the actin regulatory protein, gelsolin, is significantly reduced in primary lung tumours of patients with a smoking habit (Dosaka-Akita *et al*, 1998). Expression of the tumour suppressor p16 (INK4a) gene is often suppressed in tumour cells and p16 promoter is methylated by smoke (Kim *et al*, 2001). The components of tobacco smoke might affect D40 expression by inducing mutations of D40 promoter DNA or changes of DNA methylation and histone acetylation status. As methylation of promoter DNA leads to transcriptional repression of the genes in general, D40 promoter DNA is probably not directly methylated by agents in smoke. Nucleosome histones in D40 promoter may be acetlylated by chemicals in smoke. Transcriptional repressor or activator that binds to the D40 promoter might be inactivated or activated, respectively, by mutation of their genes or nucleosome modification of their promoters. The molecular basis of how mutagenic chemicals in tobacco smoke cause both increases and decreases in gene expression during carcinogenesis should be revealed by future studies.

As CT antigen could represent the ideal immuno-therapeutic agent against cancer, extensive studies have identified dozens of genes in the CT family. In contrast, very few studies have revealed their physiological functions or clinico-pathological characteristics of their expressions. The above information could be quite important for characterising the CT genes and when considering the treatment of cancer patients with immunotherapy. As the carboxy terminal region of D40/AF15q14 protein contains a nuclear localisation signal, it may be a nuclear protein (Hayette *et al*, 2000). This protein may play important roles in gene regulation, such as at the levels of transcription and mRNA processing. As D40/AF15q14 does not contain other conserved amino acid motifs or domains with known functions, this protein might have a veiled function in gene regulation. As individual tumour express different CT antigens, time is required to determine which CT antigen is expressed in the tumour before the patient is treated with immuno-therapy. If this could be predicted from the clinico-pathological findings of the tumours and determined quickly, treatment would be facilitated.

In summary, this study revealed that D40/AF15q14 is a new gene in cancer/testis family and its expression is significantly related to smoking. This study would contribute to further understandings of the mechanism of aberrant CT gene expression in tumours and to the application of CT genes for immunotherapy against cancer.
